# New taxa of terrestrial molluscs from Turkey (Gastropoda, Pristilomatidae, Enidae, Hygromiidae, Helicidae)

**DOI:** 10.3897/zookeys.171.2273

**Published:** 2012-02-24

**Authors:** Burçin Aşkım Gümüş, Eike Neubert

**Affiliations:** 1 Burçin Aşkım Gümüş, Gazi University, Science Faculty, Department of Biology (Zoology), Teknik Okullar, Beşevler, 06500, Ankara, Türkiye; 2 Naturhistorisches Museum der Burgergemeinde Bern, Bernastr. 15, CH-3005 Bern, Switzerland

**Keywords:** Turkey, terrestrial molluscs, new records, description of new species and subspecies

## Abstract

This paper reports on results of several collecting trips of the authors in Turkey. In the course of this research, a long-lasting question was addressed. It could be proven that the nominal species *Bulimus frivaldskyi* L. Pfeiffer, 1847 is closely related to *Meijeriella canaliculata* Bank, 1985, and thus this species is shifted from the genus *Ena* Turton, 1831, to the genus *Meijeriella* Bank, 1985. *Meijeriella canaliculata* Bank, 1985, could be recorded from Turkey for the first time. The nomenclatural situation of the species *Euchondrus septemdentatus* (Roth, 1839) vs. its replacement name *Euchondrus borealis* (Mousson, 1874) is discussed. A new arrangement of the species formely comprised in the genus *Zebrina* Held, 1837 is presented, and the genera *Rhabdoena* Kobelt & Moellendorff, 1902, and *Leucomastus* A. Wagner, 1927 are re-established. The following species and subspecies new to science could be described: *Vitrea gostelii* **sp. n.** (Pristilomatidae), *Turanena demirsoyi* **sp. n.**, *Euchondrus paucidentatus* **sp. n.**, *Rhabdoena gostelii* **sp. n**. (all Enidae), *Metafruticicola kizildagensis* **sp. n.** (Hygromiidae), and *Assyriella thospitis menkhorsti* **ssp. n.** (Helicidae). For several other species, new distribution records are listed.

## Introduction

Regarding our knowledge of the terrestrial (as well as the freshwater) malacofauna of Turkey, large areas still remain under- or even unexplored. In this work, the results originating from several independent travels and projects in Turkey are published to contribute to a more complete survey of this largely neglected part of the Turkish fauna. In the following paragraphs, the projects are briefly introduced.

The “Kaz Dağı project” (TÜBİTAK; YDBAG, 103Y110: “The Determination of the Fauna of Kaz Dağı National Park”) was performed during the years 2004 and 2005. The area is declared a National Park and is located in Northwest Anatolia between the Marmara Sea and the Aegean regions north of the Bay of Edremit with a Mediterranean climate regime. The Kaz Dağı reaches an altitude of 1745 m (39°42'16'N, 26°49'50'E). Outcrops of Palaeozoic schist can be found in the southern parts of the mountain, and limestone outcrops are widely spread in the summit area of the mountain. Gastropod specimens were collected and determined from six stations. The aim of this project was to prepare a complete faunal list in order to identify endangered species and to start with an implementation of a conservation and management plan (Gümüş 2006).

The second project was called the “Kemaliye project” (TÜBİTAK; ÇAYDAG, 105Y016: “The Determination of the Biodiversity of Kemaliye (Erzincan) and its Vicinity”) and was located in Eastern Anatolia. Kemaliye is a small village settling in the upper valley of the Euphrates (Fırat Nehri) upstream of the Keban barrage (39°15'30'N, 38°30'E, 870 m alt.). In this place, the river cuts the Anatolian plate forming a moderately deep canyon. The village is surrounded by hills of over 1600 m altitude, but the Munzur Mountains east of Kemaliye reach up to 3250 m altitude. The climate regime is typical for Central Turkey, i.e. semiarid and winter-cold, and differs considerably from that of the nearby East and Black Sea Region. Due to the high relief differences, the area offers a multitude of habitats supporting a rich fauna and flora. The scope of this project was to perform a similar survey of animal and plant species living around Kemaliye comparable to that of Kaz Dağı. The gastropod specimens were collected and identified in the years 2005 and 2007. The collections are stored in the Kemaliye Natural Museum which is being founded as an initial core of the upcoming Turkish Natural History Museum (Prof. Dr. Ali Demirsoy Doğa Tarihi Müzesi, Erzincan Üniversitesi, Kemaliye Hacı Ali Akın Meslek Yüksek Okulu; http://kemaliyemyo.erzincan.edu.tr/menuislem.php?x=47).

Another survey was conducted as a joint project between the Natural History Museum of Berne, the University of Süleyman Demirel (Isparta) and the authors in 2006. The idea was to survey some spots in the area of the large Western Anatolian Lakes around Isparta and Eğirdir and to close a gap of records along the Mediterranean coast between Fethiye and Kaş, i.e. the valley of the Eşen Çayı and the surrounding mountains. The main results of this survey will be published elsewhere, however, the taxa new to science are already presented here, because of delay in identification of some of the species collected.

### Abbreviations

BAG private collection Burçin Aşkım Gümüş, Turkey

D shell diameter

H shell height

NMBE Natural History Museum of the Burgergemeinde Bern, Switzerland

NNM Nationaal Natuurhistorisch Museum, Leiden

PD Peristome diameter

PH Peristome height

SMF Research Institute Senckenberg, Frankfurt

W number of whorls

HMT private collection H.P.M.G. Menkhorst, Krimpen op de Ijssel

All measurements mentioned are in mm.

## Results

### Family Pristilomatidae

#### Vitrea gostelii sp. n.

urn:lsid:zoobank.org:act:EE5FB4A7-8036-431F-8548-194168D16023

http://species-id.net/wiki/Vitrea_gostelii

Fig. 1

##### Type specimens.

 Holotype NMBE 23972. Type locality: Antalya Ili, Kaş, Valley of the Eşen Çayı, Palamut, 36.4088°N, 29.3644°E, 350 m alt., limestone rocks, 10 km S of Saklıkent, 06.06.2006.

##### Diagnosis.

 A small species of *Vitrea* with a spiral sculpture of small threads and faint riblets crossing the spirals.

##### Description.

 The shell colour is pale whitish, with a broadly depressed spire. The protoconch is broadly enlarged and sculptured by fine spiral threads. The whorls of the teleoconch are narrow and increase regularly, suture of medium depth. The last whorl is broadened, non-descending, well rounded. The surface of the teleoconch is sculptured by fine widely spaced riblets. The spiral sculpture of the protoconch continues on the teleoconch whorls and becomes stronger, dissecting the riblets and giving them a fine granulated appearance. On the subsurface, the riblets become faint while the spiral sculpture increases in strength.

The umbilicus is of medium width and funnel-shaped to cylindriform.

**Figure 1.**
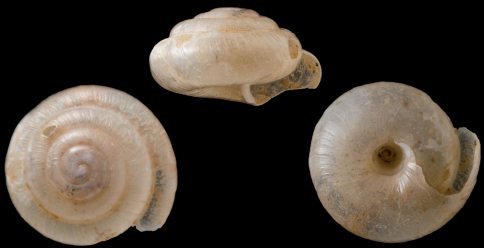
*Vitrea gostelii* sp. n., D = 2.25 mm. – All figures scaled × 20, phot H. Baur, NMBE.

##### Measurements.

 Holotype: H = 1.29; D = 2.25; PH = not measured because of damage of shell; PD = 1.05; W = 4.75.

##### Distribution

(Fig. 29): Hitherto, only the holotype is known from the type locality in the Kaş area.

##### Etymology.

 This new species is dedicated to our dear friend, the late Dr. Margret Gosteli from the Natural History Museum Berne, Switzerland. She very much enjoyed our joint visit to Turkey in 2006 and was eager to work about the malacofauna of Turkey.

##### Remark and differential diagnosis.

 The confinement of this undoubtedly new species to a pulmonate family is not easy, because only a single, dead collected shell is available. Although it looks somewhat similar to species from the family Punctidae (with *Punctum pygmaeum* (Draparnaud, 1801) known from Turkey), we have decided to place it in Pristilomatidae because of the pale color of the shell.

Under a conchological point of view, a classification within the pristilomatid genus *Lindbergia* Riedel, 1959, would also be possible, as the two genera can only be differentiated by the anatomy of their genital organs (Riedel 1959, 1960). We here confine this species to the genus *Vitrea* Fitzinger, 1833, because of its overall conchological resemblance to other species comprised in this genus. An assignment to *Oxychilus* Fitzinger is less probable, as even the smallest species of *Oxychilus* are clearly larger than the new species.

*Vitrea gostelii* sp. n. differs from all *Vitrea* species known from Turkey by the presences of riblets and a spiral sculpture. It somewhat resembles *Vitrea riedeliana*, but the spire in *Vitrea riedeliana* is more elevated. *Vitrea riedeli* has a smooth shell with a wider umbilicus with a deeper suture.

This species was found during our survey in 2006 below a large limestone rock face of probably about 50 m of height. Probably, this species lives subterraneously, and a more targeted search in the accumulated soil of carstic crevices will yield some living specimens, which then will help to corroborate the taxonomic position of this new species.

### Family Enidae. Subfamilia Buliminusinae Kobelt, 1880

#### Buliminus alepensis alepensis

(L. Pfeiffer, 1841)

http://species-id.net/wiki/Buliminus_alepensis_alepensis

Fig. 12

Helix (Cochlogena) alepi Férussac, 1821, Tabl. syst. limaçons (= “Prodome”), livr. 10: 55 (Quarto edition) [Folio edition: 59. [“Alep, côte de Syrie, au lieu dit la Coupe, à une demi-lieue de la ville”. Nomen nudum].
Bulimus alepensis L. Pfeiffer, 1841, Symbolae ad historiam heliceorum, 1: 45. Type locality: see above.

##### Specimens examined.

 Vil. Erzincan, Kemaliye, Çit Köyü, 39.1119°N, 38.6043°E, 976 m alt. 05.05.2007, leg. B. A. Gümüş, NMBE 32709/2, NMBE 32710/2 preserved; Vil. Erzincan, Kemaliye, Dilli Deresi, 39.315°N, 38.44°E, 870 m alt.,10.7.2005, leg. B. A. Gümüş, NMBE 32711/1.

##### Remark.

 This species is known to inhabit the Levant area, northern Iraq to Central eastern Turkey (see also Gittenberger and Menkhorst 1991). Kemaliye is now the northernmost record for this species. The subspecific division of this species needs further attention. We here attribute the specimens from Kemaliye to the nominotypical subspecies in accordance to Gittenberger and Menkhorst (1991).

#### Subfamilia Eninae B. B. Woodward, 1903 (1880). Tribus Enini B. B. Woodward, 1903 (1880)

##### Turanena demirsoyi sp. n.

urn:lsid:zoobank.org:act:3A9A7F78-C144-4220-B5AD-22BD0C10F0CD

http://species-id.net/wiki/Turanena_demirsoyi

Fig. 9

###### Type specimens.

 Holotype NMBE 32704; paratypes NMBE 32705/3; Vil. Erzincan, Kemaliye, Dilli Deresi, 39.315°N, 38.44°E, 870 m alt.,10.7.2005, leg. B. A. Gümüş. — Additional paratypes: SMF 312403/1, Erzincan, Kemaliye, 2.8.1987, leg. N. Kazancı, ex Slg. Neubert; Kemaliye, Yeşilyamaç (Geşo) Pass, 39.271°N, 38.567°E, 1680 m alt., 07.05.2007, leg. B. A. Gümüş, NMBE 32706/2; Kemaliye, Subatan, 39.188°N, 38.406°E, 1885 m alt., 06.05.2007, leg. B. A. Gümüş, NMBE 32707/1, NMBE 32708/4 (preserved); Vil. Erzincan, Kemaliye, Muşaga village (= Kocaçimen), 39.296°N, 38.54°E, 1375 m alt., 13.07.2005, leg. B. A. Gümüş, NMBE 32715/2.

###### Diagnosis.

 A large species of *Turanena* with a brown shell; surface of last two whorls with whitish patchy colour pattern; teleoconch sculpture of more or less distinctive riblets.

###### Description.

 Protoconch of almost 2 whorls, smooth, dome-shaped; shell colour pale brown, with a whitish patchy colour pattern on the last two whorls; shell of conical shape with an acutely ovate aperture; whorls of the teleoconch slightly rounded, increasing regularly, the whitish suture of medium depth; last whorl broadened, occupying nearly half of the height of the shell; surface of the teleoconch sculptured by more or less distinctive riblets; aperture rounded at the base with a faint white lip, light brown to dirty whitish deeper in the aperture; columellar rim of the aperture slightly reflected, a thin parietal callus connecting the apertural rims; periomphalum wide, umbilicus open, elongate, of medium width.

###### Measurements.

 Holotype: H = 12.15; D = 6.22; PH = 4.65; PD = 3.25; W = 7.

###### Etymology.

 This species is dedicated to Prof. Dr. Ali Demirsoy from the Hacettepe University in Ankara to acknowledge his outstanding contributions to Turkish zoology and support for young scientists.

###### Remarks and differential diagnosis.

 This species differs from most other *Turanena* species by its relatively large size; *Turanena tuccari* (Gittenberger, 1986) differs by its reinforced and reflected lip, which is always sharp and simple in the new species. *Turanena andronakii* (Lindholm, 1913) from the Çoruh valley and its tributaries has a more elongate slender and always completely brown shell.

###### Distribution.

 This species is only known from the surroundings of Kemaliye, its area of occupancy (AOO) is around 70km^2^.

##### Turanena cochlicopoides

E. Gittenberger & Menkhorst, 1993

http://species-id.net/wiki/Turanena_cochlicopoides

Fig. 10, 11

Turanena cochlicopoides E. Gittenberger & Menkhorst, 1993, Archiv für Molluskenkunde, 122 (Zilch Festschr.): 74, map 1, pl. 1 fig. 1 [Vilayet Gaziantep: leere Gehäuse zwischen den Felsen der Straße Gaziantep-Bahçe entlang, 60 km ö. Bahçe, in 1000 m Meereshöhe; CB 31]

###### Specimens examined.

 Vil. Erzincan, Kemaliye, between Arslanoba and Dolunay villages (the rocks around the Cahit Bilgin Park), 39.249°N, 38.599°E, 1560 m alt., 07.05.2007, leg. B. A. Gümüş, NMBE 32712/2; ditto, 07.09.2007, NMBE 32713/2.

###### Remarks.

 This is the second record for this species since its description. Interestingly, the new location is quite far away from the type locality (ca. 250 km in direct line), so it can be assumed that this species lives in a much larger area than expected before, but seems to be quite rare.

##### Tribus Multidentulini Schileyko, 1978

***Euchondrus* O. Boettger, 1883**

*Euchondrus* O. Boettger, 1883, Bericht über die Thätigkeit des Offenbacher Vereins für Naturkunde, 22/23: XX [Type species *Pupa chondriformis* Mousson, 1861 by monotypy].

###### Euchondrus septemdentatus

(Roth, 1839)

http://species-id.net/wiki/Euchondrus_septemdentatus

Figs 13–17

Pupa septemdentata Roth, 1839, Moll. spec. itinere Orientem: 19, pl. 2 fig. 2 [in insula quadam parva Oenussarum, sita inter Chium et Melaenam promontorium“; „Syriae vico quodam, dicto „Sasa“, prope Damascum“].
Bulimus triticeus Rossmässler, 1858, Iconographie der Land- und Süßwassermollusken Europa’s, 3 (5/6): 98, textfig. [«bei Jerusalem»].
Chondrus septemdentatus var. *maximus* Mousson, 1861, Vierteljahresschrift der Naturforschenden Gesellschaft Zürich, 6: 132 [nomen nudum, ms name of Bourguignat].
Chondrus septemdentatus var. *elongatus* Mousson, 1861, Vierteljahresschrift der Naturforschenden Gesellschaft Zürich 6: 132 [nomen nudum, ms name of Roth].
Chondrus septemdentatus var. *borealis* Mousson, 1874, Journal de Conchyliologie, 22: 14 [„jusqu‘à Merssina et à Tharsus“].
Buliminus septemdentatus var. *maximus* Westerlund, 1887, Fauna der in der paläarctischen Region lebenden Binnenmollusken, III: 45.
Buliminus septemdentatus var. *elongatus* Westerlund, 1887, Fauna der in der paläarctischen Region lebenden Binnenmollusken, III: 45.
Jaminia (Euchondrus) borealis , – Forcart, 1940, Verhandlungen der naturforschenden Gesellschaft Basel, 51: 202
Euchondrus borealis , – Schütt, 1983, Natur und Mensch, 1983: 57, Abb. 18.

####### Remarks.

*Euchondrus septemdentatus* (Figs 13–17) is a remarkably variable species in terms of shell shape (see also Haas 1955), and one of the most widespread taxa within the genus ranging from Southern Turkey throughout the Eastmediterranean countries almost reaching the Negev Desert (Heller 2009; the single record north of Eilat may be due to a carryover by man).

Mousson’s name *Chondrus septemdentatus* var. *borealis* (Fig. 16), which comprises the Turkish form of this species was used by Forcart (1940) as replacement name for *Pupa septemdentata* to remove the secondary homonymy with *Jaminia septemdentata* Risso, 1826 (= *Chondrina avenacea* Bruguiere, 1792). For names replaced before 1960, the rule “once a homonym, always a homonym” has to be applied (ICZN § 59). However, the replacement was never commonly accepted, and the name *septemdentatus* has always been applied for the populations of this species from Israel, the latest example for this use being Heller (2009). This results in a confusion of the correct application of the available names for this species under the condition that the specific identity is accepted. We consider Forcart‘s replacement action as invalid, because he omitted the older name *Bulimus triticeus* Rossmässler, 1858 (Fig. 14), which is also a synonym of *Pupa septemdentata*. For this reason we herewith return to use the name *septemdentatus* for this species in order to eliminate an unstable nomenclatural situation. If this point of view is not accepted, this issue has to be clarified by a ruling of the Commission following § ICZN 59.3.1.

###### Euchondrus paucidentatus sp. n.

urn:lsid:zoobank.org:act:E9CF2CD7-D99E-493C-BBC9-2AE8F83D5F7B

http://species-id.net/wiki/Euchondrus_paucidentatus

Fig. 18

####### Type specimens.

 Holotype NMBE 32688; paratypes NMBE 32689/15, coll. BANK/2; Turkey, Vil. Şanlıurfa, Nusaybin, bridge over the Çağ Çağ Deresi, 37.09°N, 41.215°E, 470 m alt., 4.8.1988, leg. E. Neubert.

####### Diagnosis.

 A medium sized species of *Euchondrus*, shell of broad conical shape, brown, dentition reduced to a parietalis, a columellaris and two palatal denticles.

####### Description.

 Shell medium sized, protoconch of 2 whorls, smooth; shell colour consistently brown; shell of broad conical shape with a rounded to semi-ovate aperture; whorls of the teleoconch well rounded, increasing regularly, the suture of medium depth with a whitish sutural line; surface of the teleoconch almost smooth, a faint sculpture of fine riblets or growth lines existing; aperture rounded, reinforced by a labial callus, apertural rims widely gaping on the parietum, reflected; dentition reduced, consisting of a cone shaped parietalis, a small columellaris, and two palatal denticles, with the lower denticle being always stronger if compared to the upper palatalis; columellar rim of the aperture widely reflected; umbilicus slit-like open, elongate.

####### Measurements.

 Holotype: H = 7.76; D = 3.74; PH = 2.95; PD = 2.4; W = 8.

####### Etymology.

 This species is called *paucidentatus* (Latin = poor in teeth) reflecting the reduced dentition of this species.

####### Remarks and differential diagnosis.

 This species is similar to *Euchondrus ledereri* (L. Pfeiffer, 1868) by its reduced dentition (Figs 19, 20), but in this species, there is always an angularis (and sometimes even an infraparietalis) present. Moreover it differs by its larger shell, which is usually white (and not brown), and the more cylindrical shape of the teleoconch. Some species of *Euchondrus* from Cyprus show a similar tendency to teeth reduction (*Euchondrus limbodentatus* (Mousson, 1854), *Euchondrus nucifragus* (L. Pfeiffer, 1848) and its subspecies, and *Euchondrus parreyssi* (L. Pfeiffer, 1846)). However, these species show an extreme development of the labial callus, which is in clear contrast to the regularly sized lip in the new species (Bank and Hovestadt 1991).

####### Distribution.

 So far, this new species is only known from its type locality.

##### Tribus Chondrulini Wenz, 1923

###### Genus Meijeriella

Bank, 1985

http://species-id.net/wiki/Meijeriella

Meijeriella Bank, 1985, Heldia 1 (2): 41.
Borlumastus Örstan & Yildirim, 2004, Basteria, 68: 126.

####### Type species.

*Meijeriella canaliculata* Bank, 1985 (by original designation).

In their paper, Örstan and Yıldırım (2004) used the following autapomorphic characters to separate their new genus *Borlumastus*: presence of a single palatal tooth, and secondly, attachment position of the penial retractor muscle.

However, presence or absence of the palatal, columellar, and parietal teeth cannot be used for characterization of genera within the family Enidae. For example, *Pseudochondrula tetrodon* (Mortillet, 1854) displays a number of variations in its apertural dentition, there are specimens without teeth as well as specimens with up to four teeth, sometimes to be found mixed up in the same population. On the other hand, browsing the species currently affiliated to *Euchondrus* Boettger, 1883 it becomes clear that in this genus, species may differ in their dentition, but still are considered to belong to the same genus. Thus it is obvious that presence or absence of a single tooth does not qualify as autapomorphic character on genus-level taxa.

The attachment position of the penial retractor muscle on the male genital system is said to differ in *Borlumastus* from that in *Meijeriella*, because in the latter genus, the muscle would attach at the epiphallus. Having investigated the genital anatomy of both species, *Meijeriella canaliculata* and *Meijeriella frivaldskyi* from Turkey it can be said that the muscles embraces the distal end of the epiphallus including a small area of the terminal part of the proximal penis section. This makes clear that there is virtually no difference in the attachment position of this muscle in all three species. For these reasons we see no argument left to keep the genus *Borlumastus*, and relegate it into the synonymy of *Meijeriella* Bank, 1985.

Remark: It has been argued that the generic name *Meijeriella* Bank, 1985 is preoccupied by *Meyeriella* Krausse, 1917 (Arch. Naturgesch., 82, A1: 95, in Hymenoptera). However, genus-level names are ruled by §56.1 and §56.2 ICZN clearly stating “Even if the difference between two genus-group names is only one letter, they are not homonyms”. Thus, *Meijeriella* has to be kept as a valid genus.

###### Meijeriella canaliculata

Bank, 1985

http://species-id.net/wiki/Meijeriella_canaliculata

Figs 2 , 4

Meijeriella canaliculata Bank, 1985, Heldia 1 (2): 42 [Greece, Mytilini (= Lesvos), at the road Keramia-Agiassos, 800 m SW Pigi Karini, approx. 39.11°N, 26.39°E].

####### Type specimens.

 Holotype NNM 55671 (not checked).

####### Specimens examined.

 Vil. Balıkesir, Edremit, Kaz Dağı, Camp Area, 39.67°N, 26.95°E, 800 m alt., 24. July 2004, leg. B. A. Gümüş, NMBE 28360/1 (preserved), NMBE 28359/1 (dry), SMF 330175/1, several specimens in coll. Gümüş.

####### Description of the genital organs

**Figure 2–3.**
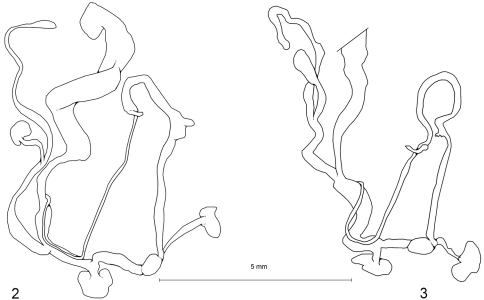
**2** Genital organs of *Meijeriella canaliculata* (Kaz Dağı) **3** Genital organs of *Meijeriella frivaldskyi* (ruins of Truva).

 (Fig. 2, shell size of dissected animal: 11.9 × 3.15 mm)**.** The morphology of the genital organs of the Turkish specimens equals that of the paratype from Lesvos shown by Bank (1985).

**Figures 4–12.**
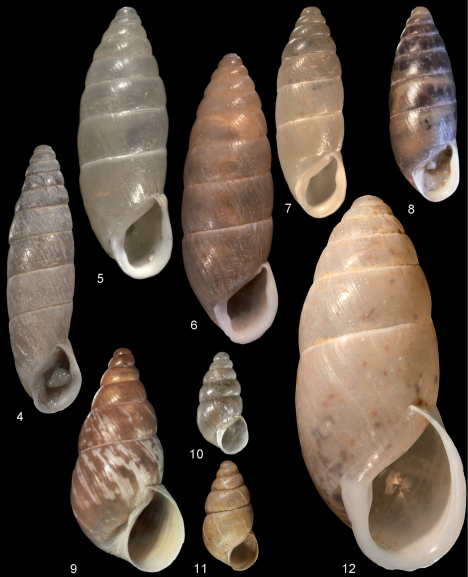
Enidae. **4–10** *Meijeriella* spp. **4** *Meijeriella canaliculata*, Vil. Balıkesir, Edremit, Kaz Dağı, Camp Area, 39.67°N, 26.95°E, H = 14.65 mm. **5–8** *Meijeriella frivaldskyi*. **5** syntype *Bulimus frivaldskyi*, SMF 63750, H = 14.8 mm **6** syntype *Bulimus raynevalianus*, MHNG 12106, H = 15.8 mm **7** *Meijeriella frivaldskyi*, SMF 312495, Ruins of Truva, leg. H.P.M.G. Menkhorst, H = 11.45 mm **8** *Meijeriella frivaldskyi*, NMBE 28357, Ruins of Truva, leg. B. A. Gümüş, 10.06.2008, H = 10.6 mm. **9–11** *Turanena* spp. **9**. *Turanena demirsoyi* sp. n., holotype NMBE 32704, Erzincan, Kemaliye, Dilli Deresi, 870 m alt., leg. B. A. Gümüş, H = 12.15 mm **10** *Turanenea cochlicopoides*, Paratype SMF 309776, Vil. Gaziantep, along the road Gaziantep → Bahçe, ca. 12 km NW Gaziantep, 1000 m alt., leg. Menkhorst, 15.07.1986, H = 5.6 mm **11** *Turanena cochlicopoides*; NMBE 32713, between Arslanoba and Dolunay villages (the rocks around the Cahit Bilgin Park), 39.249°N, 38.599°E, 1560 m alt., leg. B. A. Gümüş, 07.09.2007 **12** *Buliminus alepensis alepensis* NMBE 32709, Çit Köyü, 39.1119°N, 38.6043°E, 976 m alt. 05.05.2007, leg. B. A. Gümüş. — All figures scaled × 5.

####### Discussion.

 The shells do not differ from the illustrated holotype from the Island of Lesvos, particularly the canaliculated last whorl is clearly visible. The known distribution of this species was hitherto restricted to the island of Lesvos, where it is abundant (pers. comm. Bank 2011). This is the first record for this species from Turkey and shows that this species has a larger distribution. It mirrors the geological development of the area, since Lesvos is situated in the Eastmediterranean subduction zone and had been connected to the Turkish mainland during the Miocene.

###### Meijeriella frivaldskyi

(L. Pfeiffer, 1847)

http://species-id.net/wiki/Meijeriella_frivaldskyi

Figs 3 5–8

Bulimus frivaldskyi L. Pfeiffer, Zeitschrift für Malakozoologie, 1847, 4 (12): 191–192 [Type locality: “prope Brussa Natoliae”].
Bulimus fuscus L. Pfeiffer, Zeitschr. Malakozool., 1847, 4 (12): 192. Type locality: not given. Nomen nudum (in synonymy).
Bulimus raynevalianus Bourguignat, Amen. Malac. 1855, 29: 128, pl. 8 fig. 5–9 (shell). Type locality: “les vignes des environs de Gallipoli, oú il vit sous les mottes de terre”.
Buliminus [Brephulus] friwaldskyi f. *minor* Westerlund, 1887, Fauna pal. Reg. Binnenconch., 3: 10. Type locality: not given.
Buliminus trojanus Kobelt, Icon., 1893, (2) 6 (5–6): 81, pl. 171 fig. 1100 (shell).

####### Type locality.

 “im westlichen Theile der Troas”.

####### Type specimens.

*frivaldskyi*: Syntype SMF 63750, “Bursa”, coll. Kobelt ex L. Pfeiffer; *raynevalianus*: Syntype MHNG 12106.

####### Specimens examined.

 Vil. Çanakkale, Truva, 10.06.2003, leg. B. A. Gümüş, NMBE 28357/3 (preserved).

####### Description of the genital organs

 (Fig. 3, shell size of dissected animal: 10.6 × 3.7 mm)**.** Penis without penial appendix, subdivided in a distal and proximal section; proximal section containing a centrally perforated verge; epiphallus very long, reaching ca. 5 × the length of penis, with a small caecum at its central part, a short flagellum present; penial retractor muscle attaching at the border of penis and epiphallus; vagina as long as penis, pedunculus with a long diverticulum largely surmounting the bursa copulatrix in length.

####### Remarks.

 The principal morphological structure of the genital organs does not differ from the holotype of *Meijeriella canaliculata* (cf. Bank 1985) from the Island of Lesvos nor from that from the southern slope of the Kaz Dağı (Fig. 2). For this reason, the species *Bulimus frivaldskyi* is here confined to the genus *Meijeriella* and removed from the genus *Ena*, as already suspected by other authors (Bank 1985: 42, Hausdorf 2001). The shell of *Meijeriella frivaldskyi* differs from *Meijeriella canaliculata* by its rounded and not canaliculated last whorl; additionally, the majority of specimens of *Meijeriella frivaldskyi* differ by presence of a palatal swelling of the labium, which is said to miss in the Lesvian specimens of *Meijeriella canaliculata* (and which is also true for the Kaz Dağı population).

###### Rhabdoena

Kobelt & Moellendorff, 1902

http://species-id.net/wiki/Rhabdoena

Rhabdoena Kobelt & Moellendorff, 1902, Syst. Conch.-Cab., (1) 13 (2, 475): 1021, 1027.

####### Type species.

*Buliminus (Zebrina) caesius* O. Boettger, 1885 (= *Bulimus cosensis* Reeve, 1849) (original designation).

Until today, the genus *Zebrina* Held, 1837 comprises a dozen of species mainly from the Eastmediterranean area (Schileyko 1998). According to Bank (1988: 70), Bank and Menkhorst (1992: 126) and Bank (pers. comm. 2011), there are important differences in the anatomy of the genital organs between these species showing that this genus is very probably a paraphyletic unit. Particularly the relative position of the caecum on the epiphallus separates the species in these groups: in *Zebrina detrita* and *Zebrina fasciolata*, the epiphallar caecum is found at the terminal distal end of the epiphallus, while in all other species, the epiphallar caecum is found in a central position on the epiphallus. In the remaining group, the species of *Rhabdoena* can easily be separated from all others because of their narrow, conical, elongate shell combined with the rounded aperture, the parietal callus connecting the inclining to almost connected apertural rims, the fine riblets on the teleoconch, and the fact that they all seem to be obligate rock dwellers. The remaining group then comprises species with a broader and usually larger shell with a wide aperture, and being bottom to vegetation dwellers. This group then has to bear the generic name *Leucomastus* (type species *Leucomastus buresi* A. Wagner, 1927 = *Bulimus kindermanni* L. Pfeiffer, 1857). To illustrate the consequences, a table with some of the most important Chondrulini genera is given (Table 1).

**Table 1.** Character states in some major genera of Chondrulini (table provided by R. Bank).

**Genus**	**Penis appendix**	**Epiphallar caecum**	**Taxa**
*Thoanteus*	present	subterminal	*corneus, ferrarii, gibber, zilchi*
*Peristoma*	present	subterminal	*merduenianum, lanseum, rupestre, boettgeri*
*Caucasicola*	present	subterminal	*raddei*
*Zebrina*	present	subterminal	*detrita, fasciolata*
*Georginapaeus*	present	middle	*hohenackeri*
*Rhabdoena*	present	middle	*armenica, cosensis, mirifica, zasiensis, stokesi*
*Chondrus*	present	middle	*lycaonicus, tournefortianaus, zebra*
*Brephulopsis*	present	middle	*cylindrica, bidens, subulata, konovalovae*
*Ayna*	present	middle	*mienisi*
*Leucomastus*	present	middle	*eburnea, kindermanni, dardana?, varnensis*
*Chondrula*	absent	middle	> 10 taxa
*Mastus*	absent	middle	> 20 taxa
*Meijeriella*	absent	middle	*canaliculata, frivaldskyi, yildirimi*
*Eubrephulus*	absent	middle	*bicallosus, orientalis*

###### Rhabdoena gostelii sp. n.

urn:lsid:zoobank.org:act:9CA63D44-A467-418D-BD75-7BC254948A12

http://species-id.net/wiki/Rhabdoena_gostelii

Fig. 21

####### Type specimens.

 Holotype NMBE 33331, Vil. Erzincan, Kemaliye, Dilli Deresi, 39.315°N, 38.44°E, 870 m alt.,10.7.2005, leg. B. A. Gümüş; paratype NMBE 33332/1, Vil. Erzincan, Kemaliye, Muşaga village (= Kocaçimen 39.296°N, 38.54°E), 02.08.1987, leg. N. Kazancı, ex coll. Neubert.

####### Diagnosis.

 Shell large, protoconch dome-shaped, sculpture of fine irregularly arranged riblets, apertural rims slightly inclined, connected by a weak parietal callus.

####### Description.

 Shell large; protoconch of 2.5 whorls, smooth, dome-shaped; shell colour basically creamy whitish, with a few irregularly scattered brownish mottles; shell elongate conical with a semi-ovate aperture; whorls of the teleoconch almost flat, increasing regularly, suture of medium depth; surface of the teleoconch almost smooth, with a sculpture of fine irregularly arranged riblets on the teleoconch; apertural rims sharp, reinforced by a very weak labial callus, somewhat reflected; a shallow angularis indicated on the parietum, apertural rims slightly inclined, connected by a weak parietal callus; columellar rim of the aperture widely reflected; umbilicus slit-like open, elongate.

**Figures 13–23.**
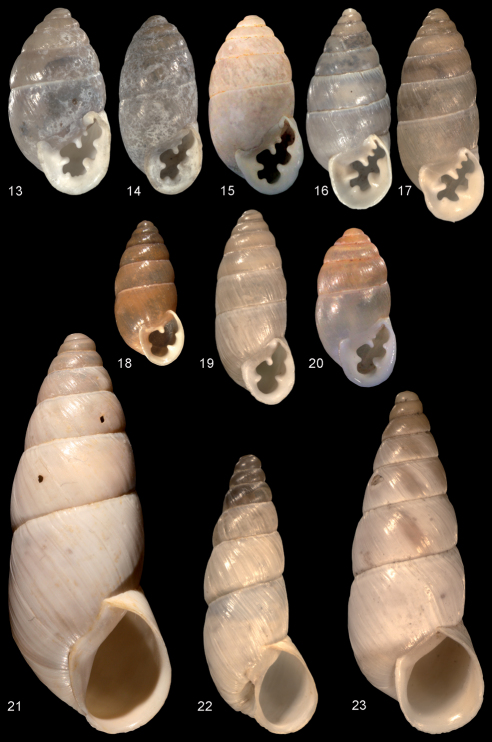
Enidae. **13–20** *Euchondrus* spp. **13** *Euchondrus septemdentatus*, syntype *Pupa septemdentata*, SMF 236888, Israel, Jerusalem, coll. Rossmässler ex Roth, Original figure from Iconographie (1), No. 922, H = 9.75 mm **14** lectotype *Bulimus triticeus* (type designation herewith based on an unpublished designation by Zilch), SMF 236889, Israel, Jerusalem, coll. Rossmässler ex Stentz, H = 9.4 mm **15** *Euchondrus septemdentatus*, NMBE 503001, Lebanon, Beirut, next to Soha water plant, 33°45.117'N, 35°45.213'E, 1474 m alt., 17.08.2008, leg. E. Neubert, H = 9.4 mm **16** syntype *Chondrus septemdentatus* var. *borealis*, ZMZ 514110, Turkey, between Mersin and Tarsus, coll. Mousson ex Schlaefli, 1861, H = 10.5 mm; **17** *Euchondrus septemdentatus*, SMF 312409, Turkey, 2.5 km SE Belen, leg. Menkhorst **18** *Euchondrus paucidentatus* sp. n., holotype NMBE 32688, Turkey, Vil. Şanlıurfa, Nusaybin, bridge over the Çağ Çağ Deresi, 37.09°N, 41.215°E, 470 m alt., 4.8.1988, leg. E. Neubert, H = 7.76 mm **19** *Euchondrus ledereri*, SMF 312412, Turkey, Meşindağı Geçidi, 15 rkm SE Eruh (= Dih), along the road to Şırnak, 37.672°N, 42.316°E, 1620 m alt., 03.08.1988, leg. Neubert, H = 10.2 mm **20** *Euchondrus ledereri*, NMBE 503488, Lebanon, Nahr Abu Ali close to Seraad, 34.283°N, 35.9288°E, 573 m alt., 19.08.2008, leg. Neubert, H = 8.32 mm **21–23** *Rhabdoena* spp. **21** *Rhabdoena gostelii* sp. n., holotype NMBE 33331, Vil. Erzincan, Kemaliye, Dilli Deresi, 39.315°N, 38.44°E, 870 m alt.,10.7.2005, leg. B. A. Gümüş, H = 20.96 mm **22** *Rhabdoena armenica*, lectotype SMF 63431, “NW Armenia (?)”, coll. Nägele ex Tembé, H = 14.6 mm **23** *Rhabdoena cosensis*, lectotype *Buliminus (Zebrina) caesius* O. Boettger, 1885, SMF 14485, Turkey, “Smyrna” [= Izmir], coll. O. Boettger ex Goldfuss, H = 17.9 mm. -- All figures scaled × 5.

####### Measurements.

 Holotype: H = 20.96; D = 8.56; PH = 7.14; PD = 5.4; W = 8.5.

####### Etymology.

 This new species is dedicated to our dear friend, the late Dr. Margret Gosteli from the Natural History Museum Berne, Switzerland.

####### Distribution.

 This species is only known from a very small range in the area of Kemaliye.

####### Remarks and differential diagnosis.

 This new species is currently confined to *Rhabdoena* because of the overlap of conchological characters with other species of this subgenus. The new species differs from all other known species by its size (it is the largest species in the subgenus). The conchologically closest taxon is *Rhabdoena cosensis* (Reeve, 1849) (Fig. 23), which has a more conical shell, a more mammillate protoconch, a more open umbilicus, and a much smaller last whorl. The other Turkish species, *Rhabdoena armenica* (Nägele, 1903) (Fig. 22), is much smaller, has a widely open umbilicus, and a more rigid mode of ribbing. The latter species is known from two lots in the collection of SMF only, and has not been found back until now. The second lot in SMF (not illustrated here) is said to originate from Tokat (coll. Nägele). Probably, this species is a local endemic species with a restricted distribution area like *Rhabdoena gostelii* sp. n.

### Family Hygromiidae

#### Metafruticicola kizildagensis sp. n.

urn:lsid:zoobank.org:act:FDBFEEA4-D4C8-40CB-A47D-9BB7B8D8780C

http://species-id.net/wiki/Metafruticicola_kizildagensis

Fig. 24

##### Type specimens.

 Holotype NMBE 32690; paratypes NMBE 32691/3, coll. Gümüş/5; Turkey, Vil. Isparta, Şarkikaraağaç, Kızıldağ, ca. 5 km SE of Şarkikaraağaç, 38.0403750°N, 31.3653850°E, 1500 m alt., 24.12.2005, leg. B. A. Gümüş.

##### Diagnosis.

 A large species of *Metafruticicola*, depressed white shell with a single brown spiral band below the periphery, last whorl bluntly angled, surface of teleoconch with irregular axial stripes, smooth, umbilicus open.

##### Description.

 Protoconch of 2.25 whorl, sculptured by small radial riblets with interspersed pits, white to pale yellow; teleoconch of 4 whorls, basic colour white, with two partly fused brown spirals on the upper surface, and a single brown spiral band below the periphery; shell depressed, spire only slightly elevated, last whorl bluntly angled; surface of teleoconch with irregularly arranged axial stripes, smooth; last whorl only slightly descending below the shell‘s periphery; aperture depressed oval, reinforced by a weak labial callus, peristomial rim slightly reflecting over the umbilicus; umbilicus open, initially cylindrical, with the last whorl somewhat eccentrically increasing.

**Figures 24–26.**
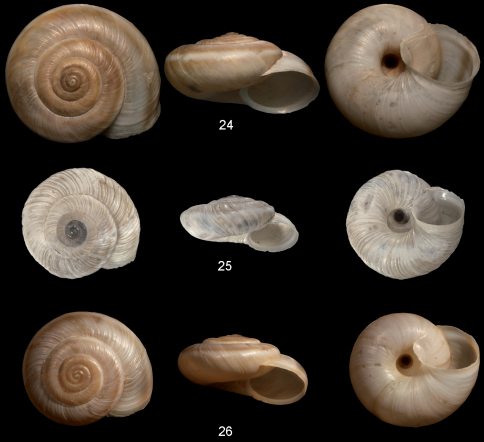
*Metafruticicola* spp. **24**. *Metafruticicola kizildagensis* sp. n., holotype NMBE 32690 Vil. Isparta, Şarkikaraağaç, Kızıldağ, ca. 5 km SE of Şarkikaraağaç, 38.0403750°N, 31.3653850°E, 1500 m alt., 24.12.2005, leg. B. A. Gümüs, D = 20.44 mm **25** *Metafruticicola dedegoelensis*, Vil. Isparta, Dedegöl Dağı, Yenişarbademli, Alma Uşağı Mevki, 2350 m alt., 26.05.2002, leg. B. A. Gümüş, D = 15.5 mm **26** *Metafruticicola oerstani*, Isparta, Barla Dağı, southern slope, 2000 m alt., 38.02°N, 30.7°E, NMBE 23902. — All figures scaled × 2.

##### Measurements.

 Syntype: H = 10.8; D = 20.44; PH = 5.65; PD = 10.3; W = 6.25.

##### Etymology.

 This species is named after the Kızıldağ mountain NE of the city of Isparta.

##### Distribution.

 This species is only known from its type locality. However, we assume that as is the case in the other *Metafruticicola* species mentioned above, this species may locally be present in the alpine to subalpine region of the inner Anatolian mountain chain.

##### Remarks and differential diagnosis.

 This species is considerably larger than the two species of *Metafruticicola*, *Metafruticicola dedegoelensis* Hausdorf et al., 2004 (Fig. 25), and *Metafruticicola oerstani* Hausdorf et al., 2004 (Fig. 26), which live nearby. The shell of *Metafruticicola dedegoelensis* differs by its shell sculptured with strong ribs. The shell of *Metafruticicola oerstani* is smaller, more depressed with short, bristle-like hairs on the teleoconch surface, which usually are lost in adult shells but still can be found on the umbilicus walls, and scattered hair scars on the teleoconch surface. Moreover, its umbilicus is wider and more perspective if compared to *Metafruticicola kizildagensis* sp. n., which has no hairs nor hair scars and a narrower and more cylindrical umbilicus.

### Family Helicidae

#### Assyriella thospitis menkhorsti ssp. n.

urn:lsid:zoobank.org:act:C94D7CB6-0483-4B41-BA38-0355A52AD6C8

http://species-id.net/wiki/Assyriella_thospitis_menkhorsti

Fig. 28

##### Type specimens.

 Holotype NMBE 33333, paratypes NMBE 16599/3, Turkey, Bitlis, Kireçtaşı, quarry, 38.37°N, 42.1°E, 9.5.2011, leg. & ex coll. Menkhorst; additional paratypes HMT/16.

##### Additional specimens examined.

 Bitlis 1.8 km NE. Kokarsu, 1723 m alt., 38.3942°N, 42.2685°E, 10.5.2011, HMT/6.

##### Diagnosis.

 Stronge rib sculpture on the teleoconch, presence of greenish-yellowish periostracum and a columellar ridge on the basal apertural rim.

##### Description.

 Large shells, depressed to slightly elevated spire; protoconch with 1.5 flat and slightly ribbed whorls; last whorl strongly descending at the aperture; teleoconch sculpture of regular axial riblets; basic shell colour greenish to yellowish due to the periostracum; spiral bands bluish; aperture cross-oval, reinforced by a labial callus, moderately reflected; umbilicus slit-like open to sometimes closed; columellar labial callus or ridge present.

**Figures 27–28.**
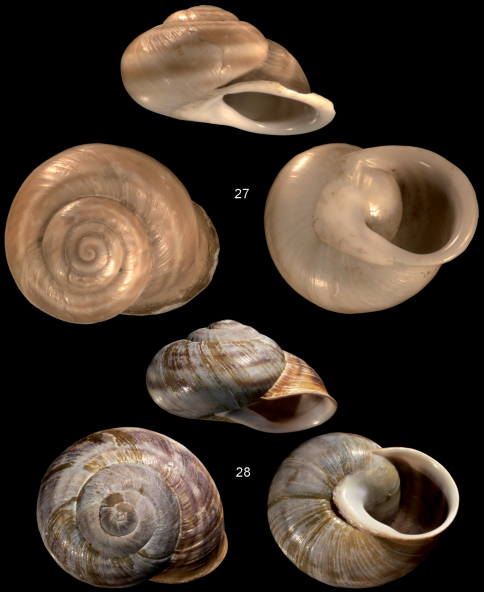
ssp. Figure **27** *Assyriella thospitis thospitis*, holotype NNM 56804, Bitlis, Kermate, 10 km SW of Setek, 1550 m alt., May 1990, leg. Neuteboom, D = 37.6 mm **28** *Assyriella thospitis menkhorsti*ssp. n., holotype NMBE 33333, Bitlis, Kireçtaşı, quarry, 38.37°N, 42.1°E, 9.5.2011, D = 33.46 mm. — All figures scaled × 1.5.

##### Measurements.

 Holotype: H = 19.8; D = 33.46; PH = 7.2; PD = 21.6; W = 4.75.

##### Etymology.

 This new subspecies is named in honor of Dipl. Ing. H. P. M. G. Menkhorst, a keen malacologist, to acknowledge his deep interested in and outstanding contributions to the knowledge of the biodiversity of the Turkish malacofauna.

##### Distribution.

*Assyriella thospitis menkhorsti*ssp. n. lives in a small area southeast to the range of the nominotypical subspecies (Schütt and Subai 1996: 118, 141).

**Figure 29.**
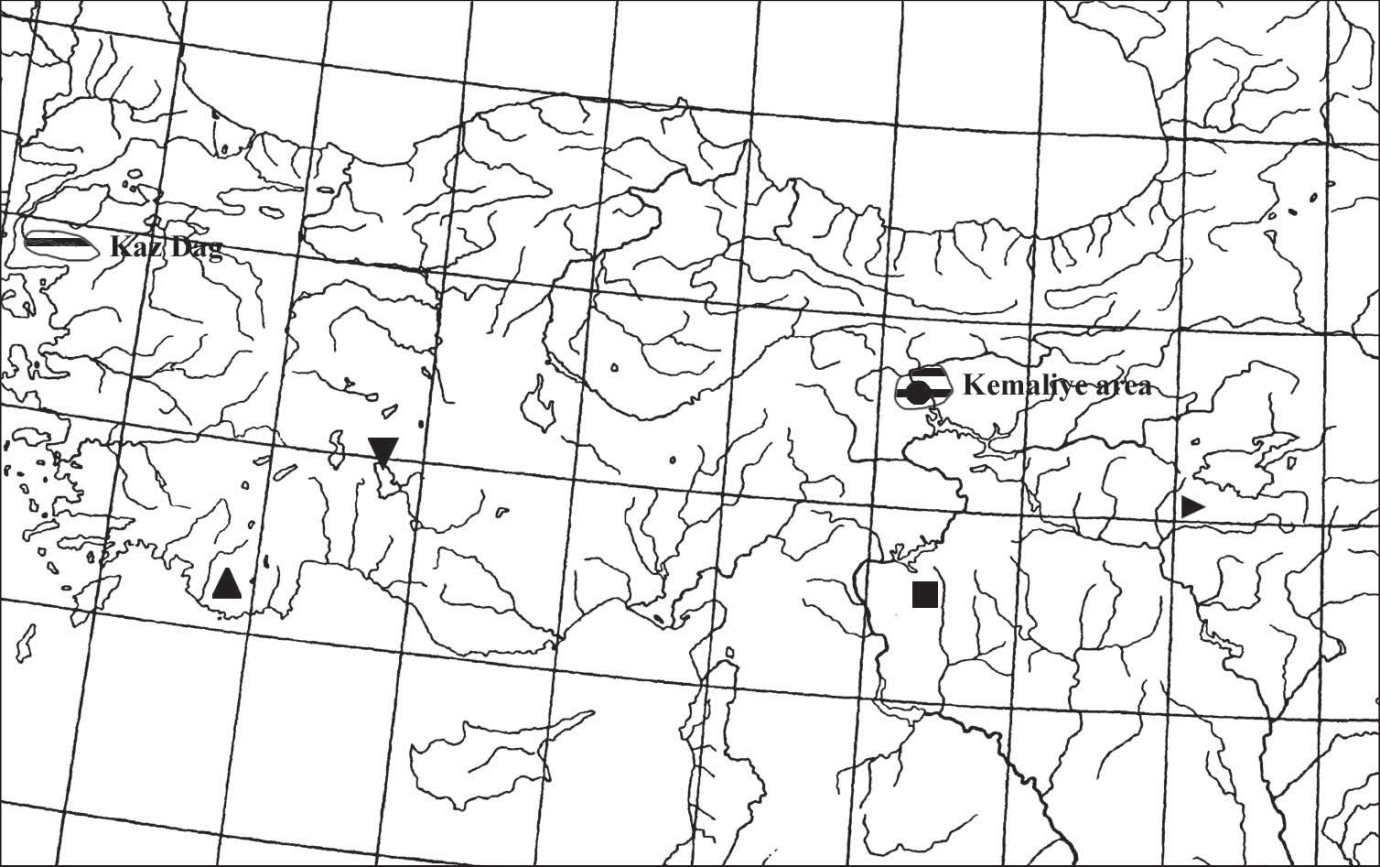
Distribution map. ▲ *Vitrea gostelii* sp. n. ● *Turanena demirsoyi*sp. n. and *Rhabdoena gostelii*sp. n. ■ *Euchondrus paucidentatus* sp. n. ▼ *Metafruticicola kizildagensis* sp. n. ► *Assyriella thospitis menkhorsti* ssp. n.

##### Remarks and differential diagnosis.

 This subspecies differs from the nominotypical subspecies in several character states (Fig. 27). The main character states are the stronger rib sculpture on the teleoconch, the bluish spiral bands (brown in *Assyriella thospitis thospitis* Schütt & Subai, 1996), the greenish-yellowish periostracum (almost transparent and colourless in *Assyriella thospitis thospitis*), the usually slit-like umbilicus (always closed and thickly calloused in *Assyriella thospitis thospitis*), presence of a ± well developed columellar ridge on the basal apertural rim.

## Supplementary Material

XML Treatment for Vitrea gostelii

XML Treatment for Buliminus alepensis alepensis

XML Treatment for Turanena demirsoyi

XML Treatment for Turanena cochlicopoides

XML Treatment for Euchondrus septemdentatus

XML Treatment for Euchondrus paucidentatus

XML Treatment for Meijeriella

XML Treatment for Meijeriella canaliculata

XML Treatment for Meijeriella frivaldskyi

XML Treatment for Rhabdoena

XML Treatment for Rhabdoena gostelii

XML Treatment for Metafruticicola kizildagensis

XML Treatment for Assyriella thospitis menkhorsti
